# Zieve’s Syndrome: An Underdiagnosed Cause of Non-immune Hemolytic Anemia

**DOI:** 10.7759/cureus.52034

**Published:** 2024-01-10

**Authors:** Rui Ribeiro, Marli Ferreira, Rui Coelho, Cláudia Pereira

**Affiliations:** 1 Internal Medicine, Centro Hospitalar Universitário do Porto, Porto, PRT

**Keywords:** hemolytic anemia, acute alcoholic hepatitis, jaundice, alcohol liver disease, zieve’s syndrome

## Abstract

Zieve’s syndrome is an underdiagnosed condition characterized by the triad of jaundice, hemolytic anemia, and hyperlipidemia in the setting of chronic alcohol use. It may be accompanied by acute alcoholic hepatitis. The distinction between the coexistence of acute alcoholic hepatitis with Zieve’s syndrome and Zieve's syndrome in isolation is crucial, given the different treatments and prognoses in these situations.

A 35-year-old woman presented with complaints of abdominal discomfort, nausea, and vomiting in the previous week. She was a heavy drinker with resultant cirrhosis, splenomegaly, and esophageal varices. An ancillary test showed hemolytic anemia, moderately elevated transaminases, hyperbilirubinemia, and coagulopathy. A negative direct Coombs test established the anemia as non-immune, supporting the diagnosis of Zieve’s syndrome despite the absence of hyperlipidemia. Maddrey’s discriminant function score was 92 points, so she was treated with supportive measures, as well as corticosteroids in the setting of acute alcoholic hepatitis. The patient showed a favorable clinical and analytical evolution and was discharged home one month following admission with her hemoglobin levels stabilized.

Previous literature focused on the distinction between Zieve's syndrome and acute alcoholic hepatitis but they may coexist.

## Introduction

First described in 1958 by Dr. Leslie Zieve, Zieve’s syndrome presents as a triad of hemolytic anemia, jaundice, and transient hyperlipidemia that develops secondary to alcohol-induced liver injury [1]. It is underdiagnosed probably due to the lack of awareness within the medical community. Therefore, its true prevalence is unknown [2].

Probably due to its transient nature, hyperlipidemia is not apparent at the time of diagnosis in some patients [1].

Anemia is a common finding in alcoholic liver disease and can be explained by nutritional deficits, medullary alcohol toxicity, chronic inflammation, hypersplenism, or hemolysis in the context of Zieve’s syndrome [2]. The pathogenesis of Zieve’s syndrome remains poorly understood [2]. Abstention from alcohol and supportive management are the basis of its treatment [2].

Jaundice and hyperbilirubinemia are common findings in both Zieve's syndrome and acute alcoholic hepatitis [2]. Previous literature focused on the distinction between these two entities [2] but they may coexist.

## Case presentation

A 35-year-old Caucasian woman with a history of chronic alcohol abuse with resultant cirrhosis and portal hypertension manifestations (small esophageal varices and splenomegaly) sought medical attention at the emergency service with complaints of an increased abdominal perimeter with diffuse abdominal pain, nausea, and vomiting for one week prior to admission. She denied fever, diaphoresis, and gastrointestinal blood loss and was medicated with propranolol, spironolactone, and lactulose. The patient had been evaluated three months earlier in the outpatient clinic, and ascites was not present. Bilirubin levels were 3.79 mg/dL, with hemoglobin of 10.5 g/dL and platelet count of 102 per uL.

She admitted to drinking alcohol in the days before coming to the hospital. Physical examination revealed blood pressure of 118/68 mmHg, tympanic pressure of 37ºC, generalized jaundice, moderate volume ascites, mild edema of the lower limbs, and signs of minimal encephalopathy with an abnormal retain test. A digital rectal exam was not suggestive of digestive blood loss. The chest radiograph and electrocardiogram were unremarkable. The abdominal ultrasound with Doppler excluded portal vein thrombosis, dilation of the intra and extrahepatic bile ducts, and focal lesions. Cytological and microbiological evaluation of ascitic fluid ruled out spontaneous bacterial peritonitis.

**Figure 1:**
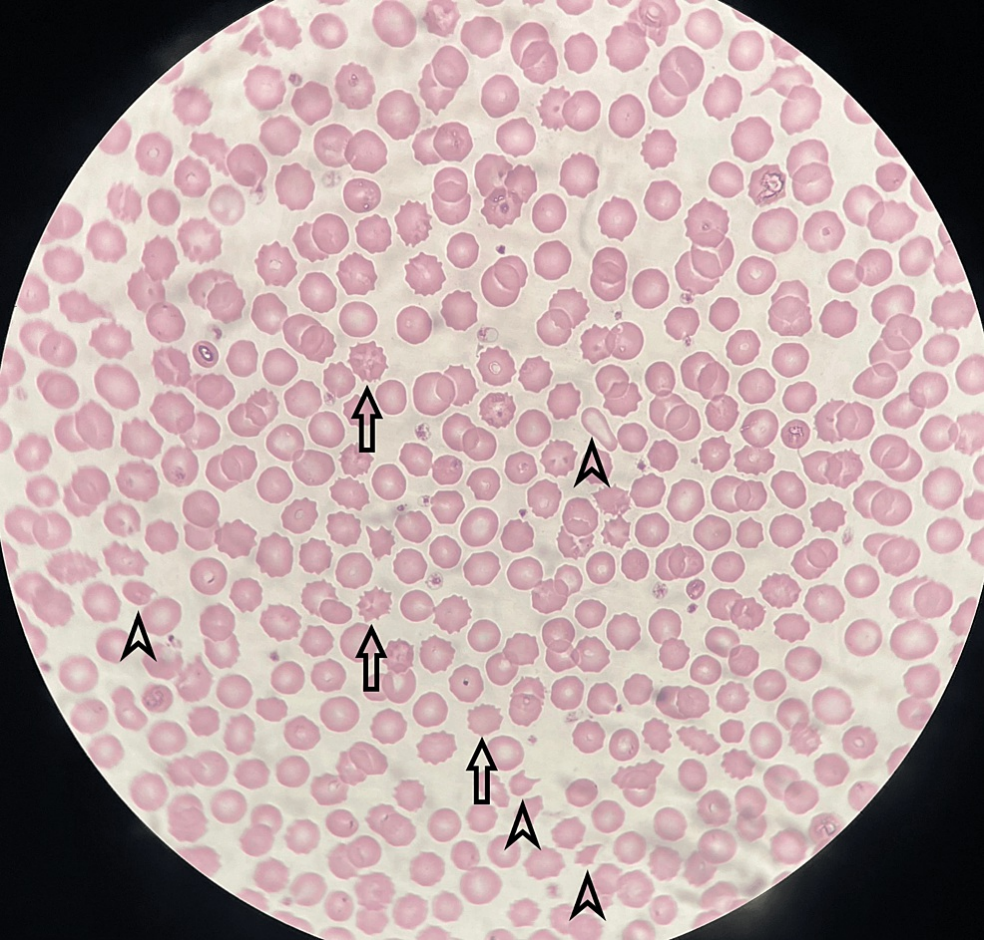
Peripheral blood smear showing acanthocytes (arrows) and schistocytes (arrowheads).

A complete blood count revealed severe anemia with a hemoglobin value of 6 g/dL. Lactate dehydrogenase was increased, and the haptoglobin level was untraceable. The peripheral blood smear showed schistocytes and acanthocytes (Figure 1), and the direct Combs test was negative. The lipid panel and pancreatic enzymes were normal. Prolonged prothrombin time was also detected. Other notable laboratory studies on admission are summarized in Table 1.

**Table 1:** Laboratory studies on admission.

	Reference range	Admission
Hemoglobin, g/dL	12-15	6
Hematocrit, %	36-46	18.6
Mean corpuscular volume, fL	83-101	117
Red cell distribution width, %	11.5-14.5	15.8
Reticulocyte, %	0.5-2.5	11.62
Platelet count, per uL	150000-400000	59000
Total protein, g/dL	6-7.3	6.78
Albumin, g/dL	3.5-5	2.43
Total bilirubin, mg/dL	0.2-1.1	9.54
Direct bilirubin, mg/dL	0.0-0.3	6.61
Alkaline phosphatase, U/L	35-104	213
Aspartate aminotransferase, U/L	10-30	189
Alanine aminotransferase, U/L	10-36	59
Prothrombin time, seconds	11,4	29.5
Creatinine, mg/dL	0.5-0.9	1.87
Cholesterol, mg/dL	0-200	118
Triglycerides, mg/dL	35-135	101
Lactate dehydrogenase, U/L	135-214	1033
Folic acid, ng/mL	3.9-26.8	2
B12 vitamin, pg/mL	191-663	867
Iron, ug/dL	50-150	80
Lipase, U/L	30-190	53
Amylase, U/L	0-53	3

An initial concern about the possibility of thrombotic thrombocytopenic purpura was raised based on the fact that this patient was a young woman with hemolytic anemia, thrombocytopenia, and neurological abnormalities but the patient had a low PLASMIC score (three points), and later ADAMTS13 activity testing ruled out the diagnosis. She was diagnosed with Zieve’s syndrome in the setting of an acute-on-chronic liver failure triggered by acute alcoholic hepatitis (Maddrey discriminant function score of 92 points and Model for End-Stage Liver Disease-Na score of 32 points). She started supportive treatment (blood transfusion, vitamin supplements, diuretics, and prevention of alcohol withdrawal) and a four-week course of prednisolone targeting alcoholic hepatitis. The patient’s symptoms improved slowly. Her hemoglobin levels remained stable with a subsequent decrease in bilirubin levels and normalization of renal function. She was discharged home one month after admission with subsequent follow-up as an out-patient.

## Discussion

Zieve’s syndrome’s essential clinical features are jaundice, hyperlipidemia, and hemolytic anemia. This illness follows excessive drinking and improves once the drinking stops [1]. Although it is a rare disease, some studies estimate its frequency in a general medical ward to be one in 1600 admissions [3].

The pathogenesis of this syndrome has yet to be fully understood. It is hypothesized that hyperlipidemia results from a massive fat mobilization to or from the liver [1] and that high levels of lysolecithin and lysocephalin could trigger erythrocyte destruction [4]. It was also shown that alcohol-induced vitamin E deficiency of erythrocytes leads to a decrease in membrane-bound polyunsaturated fatty acid, which may destabilize pyruvate kinase and increase the susceptibility to hemolysis [5]. Jaundice and hyperbilirubinemia seem to result from hemolysis and mostly from alcohol-induced intrahepatic cholestasis.

There are few reports referring to the coexistence of Zieve’s syndrome and acute alcoholic hepatitis [6,7]. This difference is critical to notice since the two illnesses have different prognoses: most patients with Zieve’s syndrome will recover four to six weeks after alcohol withdrawal, while a significant percentage of patients with acute alcoholic hepatitis may progress to liver failure and death and may benefit from treatment with corticosteroids. The distinction is not always easy, since both diseases can lead to high bilirubin levels (mainly direct bilirubin). Hyperbilirubinemia secondary to hemolysis may misguide clinicians to overtreat with steroids based on a high Maddrey’s discriminant function score leading to an increased risk of infection. Thus, it is important to weigh the impact of this score's parameters (hyperbilirubinemia and coagulopathy) on the final value. In the present case, the patient had severe coagulopathy that justified treatment with steroids.

This patient presented with moderately elevated transaminases, an aspartate aminotransferase/alanine aminotransferase ratio of more than 2, direct bilirubin higher than 6 mg/dL, and coagulopathy in the presence of a long history of heavy alcohol use and a very high value of Maddrey score, so it was assumed she had acute alcoholic hepatitis. The finding of anemia associated with low haptoglobin levels, high lactate dehydrogenase levels, and schistocytes in the peripheral blood smear led to the diagnosis of hemolytic anemia. A negative direct Coombs test and the latter ADAMTS13 activity test raised suspicion of Zieve’s syndrome. The patient presented with normal cholesterol and triglycerides serum levels. As stated before, hyperlipidemia is one of the features of this syndrome. However, half of the patients with Zieve’s syndrome have been found to have no lipemia [1]. This may be because the rise in serum lipids is transient and often precedes the onset of hemolysis [6]. Additionally, this rise appears less extensive in patients with more advanced cirrhosis [1], as with this patient. There have been other recent cases reported with normal lipid profiles [8].

Despite the absence of hyperlipidemia and the presence of a concomitant cause of hyperbilirubinemia, the patient was diagnosed with Zieve’s syndrome in the setting of non-immune hemolytic anemia and recent alcohol abuse.

There is no specific treatment for Zieve’s syndrome. Supportive care remains the standard therapy, and recovery within four to six weeks following alcohol cessation is documented in case reports [7].

## Conclusions

The presence of hemolysis in patients with alcohol-induced liver injury should raise suspicion for Zieve’s syndrome. Hyperlipidemia is one of the typical features of this syndrome but it may be transient and not apparent at the time of diagnosis. Its absence should not exclude the diagnosis in the right clinical setting.

Previous literature focused on the distinction between Zieve's syndrome and acute alcoholic hepatitis but they may coexist. This case report exemplifies that occurrence.
